# Clinical impact of PTEN methylation status as a prognostic marker for breast cancer

**DOI:** 10.1186/s43141-021-00169-4

**Published:** 2021-05-10

**Authors:** Amal Ramadan, Maha Hashim, Amr Abouzid, Menha Swellam

**Affiliations:** 1 Biochemistry Department, Genetic Engineering and Biotechnology Research Division, National Research Centre, El-Bohouth Street, Dokki, Giza, 12622 Egypt; 2 High Throughput Molecular and Genetic Laboratory, Center for Excellence for Advanced Sciences, National Research Centre, Dokki, Giza, Egypt; 3 Surgical Oncology Department, Mansoura Oncology Centre, Faculty of Medicine, Mansoura University, Mansoura, Egypt

**Keywords:** Breast cancer, PTEN methylation, clinicopathological factors, CEA, CA15.3

## Abstract

**Background:**

Aberrant DNA methylation of phosphatase and tensin homolog (PTEN) gene has been found in many cancers. The object of this study was to evaluate the clinical impact of PTEN methylation as a prognostic marker in breast cancer. The study includes 153 newly diagnosed females, and they were divided according to their clinical diagnosis into breast cancer patients (*n* = 112) and females with benign breast lesion (*n* = 41). A group of healthy individuals (*n* = 25) were recruited as control individuals. Breast cancer patients were categorized into early stage (0–I, *n* = 48) and late stage (II–III, *n* = 64), and graded into low grade (I–II, *n* = 42) and high grade (III, *n* = 70). Their pathological types were invasive duct carcinoma (IDC) (*n* = 66) and duct carcinoma in situ (DCI) (*n* = 46). Tumor markers (CEA and CA15.3) were detected using ELISA. DNA was taken away from the blood, and the PTEN promoter methylation level was evaluated using the EpiTect Methyl II PCR method.

**Results:**

The findings revealed the superiority of PTEN methylation status as a good discriminator of the cancer group from the other two groups (benign and control) with its highest AUC and increased sensitivity (96.4%) and specificity (100%) over tumor markers (50% and 84% for CEA and 49.1% and 86.4% for CA15.3), respectively. The frequency of PTEN methylation was 96.4% of breast cancer patients and none of the benign and controls showed PTEN methylation and the means of PTEN methylation (87 ± 0.6) were significantly increased in blood samples of breast cancer group as compared to both benign and control groups (25 ± 0.7 and 12.6 ± 0.3), respectively.

Methylation levels of PTEN were higher in the blood of patients with ER-positive than in patients with ER-negative cancers (*P* = 0.007) and in HER2 positive vs. HER2 negative tumors (*P* = 0.001). The Kaplan-Meier analysis recognizes PTEN methylation status as a significant forecaster of bad progression-free survival (PFS) and overall survival (OS), after 40 months follow-up.

**Conclusions:**

PETN methylation could be supposed as one of the epigenetic aspects influencing the breast cancer prognosis that might foretell more aggressive actions and worse results in breast cancer patients.

## Background

Breast cancer (BC) is considered as the most mutual cancer-related death amongst women universally [1]. Thus, searching for the contribution of gene expression in profiling the clinical subgroups and their evaluation as prognostic factors is of great significance in the prediction of disease outcome.

Breast carcinogenesis is the progressive rise of genetic modifications involving point mutations, deletions, oncogene activation, or the inactivation of tumor suppressor [2]. Epigenetics is inherited alterations in gene expression without changes in DNA sequence and has received a great interest in the last decade [3]. Epigenetic variations that happen in malignant transformation include DNA methylation alterations, involving overall hypomethylation, pivotal hypermethylation, histone mutations, and nucleosomal recasting [4].

DNA hypermethylation mechanism is an epigenetic DNA change that generally occurs in approximately 70–80% of CpG sites in the human genome by the methyl group adding to cytosine residues of the CpG dinucleotides (CpG) [5]. So, DNA methylation contributes in the gene activity regulation and transcription with no changes in the fundamental nucleotide sequence of the genome [6]. CpG islands are often located within the promoter regions of genes, and their hypermethylation can lead to tumor suppressor genes inactivation that is determined in many tumors with transcriptional quieting mechanism, while gene activation may result to CpG hypomethylation [7]. Thus, tumor suppressor genes silencing with hypermethylation in promoter zones and activation of oncogenes or pro-metastatic genes with hypomethylation are trademarks of an initial molecular marker for tumor cells and cancer evolution. Patterns of DNA methylation in applicant genes may be precious for the initial discovery and forecast of cancer and also is beneficial for drug response prediction in patients [7].

Breast cancer gene 1 (BRCA1), retinoic acid receptors-b2 (RAR-b2), and RASSF1A are examples of tumor suppressor genes, whose loss of expression in BC is partially attributed to promoter hypermethylation [8–11]. BRCA1 promoter methylation was more frequent in invasive than in in situ carcinomas and also was positively associated with mortality in breast cancer [10]. Methylation levels of RARb2 and RASSF1A increased significantly during the progressive stages of BC development, and their hypermethylation was associated with unfavorable features of BC. So they can be used as malignant potential predictors [11].

Phosphatase and tensin homolog (PTEN) located at the 10q23 region has been reported as tumor suppressor gene mutated in many human cancers [12]. A variety of essential processes was regulated by PTEN such as translation, cell cycle series, and programmed cell death by obstructing the serine/threonine kinase Akt/PKB triggering. Its expression was noticed to be reduced or lost in many human tumors, including lymphoid neoplasia [13], brain tumors [14], hepatocellular carcinomas [15], melanomas [16], thyroid carcinomas [17], endometrial carcinomas [18], and breast carcinomas [19, 20].

PTEN inactivation has been deemed to happen by hypermethylation of its promoter in BC (breast cancer) leading to unfortunate gene silencing [21–23]. Also, PTEN promoter hypermethylation in BC reported notably diverse rates. These diverse results of such studies [21–24] do require further assessment of the relevance between the degree of PTEN promoter hypermethylation and breast cancer which might yield a worthy marker for early detection and help in knowing how these alterations affect the disease progression and prognosis for the patient.

DNA methylation has been widely studied in breast disease patients in tissue or formalin-fixed paraffin block sections (FFPE) (benign breast lesion and cancer groups) and revealed a significant difference between both groups [25–27]. Thus, the detection level of methylation in blood samples will be of great value to estimate their value as a minimal non-invasive diagnosis as previously reported [28–31] and prognostic marker.

This study aimed to quantitatively assess the levels of methylation in the promoter region of the PTEN gene in blood samples of breast diseased groups (benign breast lesion and cancer groups), and besides, healthy females were recruited as controls, using EpiTect Methyl II PCR. In addition, we aimed to evaluate the association of methylation level of this gene with clinico-pathological factors and survival outcomes and compare between the prognostic significance of PTEN gene methylation and tumor markers among breast cancer patients.

## Methods

### Study design and sample processing

After obtaining an approval for the study, a total of 153 individuals whom fulfilled the inclusion criteria and signed their informed consent were enrolled in the study. Patients were categorized according to their clinicopathological criteria into newly diagnosed breast cancer group (*n* = 112) (classified into 66 patients with IDC and 46 patients with DCI) and patients with benign breast lesions (*n* = 41). A group of healthy females (*n* = 25) were recruited as control individuals. Clinical and demographic factors were reported in Table 1. Staging and grading for breast cancer patients were evaluated according to previously reported criteria [32, 33]. The eligibility criteria were those who did not undergo any treatment protocol or have any other malignant tumor and patients who did not meet these criteria were removed from the study. Five millimeters of blood was therefore obtained from the enrolled individuals and separated into two tubes; for evaluation of tumor markers, 2.5 mL was put in polymer gel and clot activator tube, then serum was separated and stored in − 80 °C until the determination of tumor markers, and the remaining 2.5 mL blood was placed in the other containing EDTA tube for further processing to detect PTEN methylation as previously reported for isolation of DNA from whole blood to detect methylation status [28–31].

**Table 1:** Clinical and demographic characters for enrolled patients

Factors	Breast cancer (*n* = 112)	Benign breast lesion (*n* = 41)	Control group (*n* = 25)
Age (mean ± SEM)	53 ± 0.8	50 ± 1.24	49 ± 1.6
≤ 50 years (*n* = 94)	58	21	15
> 50 years (*n* = 84)	54	20	10
Menopausal status			
Pre-menopause (*n* = 114)	69	27	18
Post-menopause (*n* = 64)	43	14	7
Pathological type			
DCIS	46		
IDC	66		
Clinical stage			
Early stage	48		
Late stage	64		
Histological grade			
Low grade	42		
High grade	70		
Lymph node involvement			
Negative	60		
Positive	52		
ER status			
Negative	60		
Positive	52		
PgR status			
Negative	58		
Positive	54		
HER-2neu status			
Negative	40		
Positive	72		

### Hormonal receptor status examination

The immunohistochemistry method was used to examine the expression of hormonal receptors: estrogen receptor (ER), progesterone receptor (PgR), and human epidermal growth factor receptor-2 (HER-2/neu) [34, 35].

### Tumor marker assessment

CEA and CA15.3 were assessed in serum samples using a commercial ELISA (enzyme-linked immunosorbent assay) kit (Immuno-speccorporation, Netherlands) following the guidelines in the manufacturer’s instructions. Their concentrations were measured using GloMax_®_-Multi detection system (Promega, USA).

### Detection of methylation status using EpiTect Methyl II PCR system

The EpiTect Methyl II PCR system employs a method that is built on the detection of residual input DNA after cleavage with the restriction enzyme, including a methylation-sensitive which digests unmethylated DNA and a methylation-dependent that will digest methylated DNA. Next to digestion, real-time PCR is used to quantify the residual DNA in each enzyme reaction, using primers that border a promoter (gene) region of concern. This technique is conveyed out through three stages: withdrawal of DNA from blood, restriction digestion of extracted DNA, and quantification of methylation grade using the QPCR method.

### Extraction of DNA from whole blood samples

Extraction of DNA was performed according to the manufacturer’s protocol using QIAamp DNA mini blood kit (Cat No # 51104, Qiagen, Germany). Nano-drop spectrophotometer (Quawell, Q-500, Scribner, USA) was used to detect the purity and the concentration of extracted DNA then kept at – 20 °C till restriction digestion step.

### Restriction digestion

The EpiTect Methyl II DNA Restriction Kit (cat. no. 335452) was used to do restriction digestion of extracted DNA. Genomic DNA was divided equally into four parts and exposed to mock (no enzyme [M0]), methylation-sensitive (MSRE [Ms]), methylation-dependent (MDRE [Md]), and double (MSRE and MDRE [Msd]) restriction endonuclease digestion. The reactions were incubated at 37 °C for 6 h in a thermal cycler (SureCycler 8800, Agilent, Santa Clara, CA, USA). After incubation, the reactions were ended by heat-inactivating the enzymes at 65 °C for 20 min. After that, the enzyme reactions were kept at – 20 °C till the time of Q-PCR performance.

### Assessment of methylation status using QPCR

**Fig. 1:**
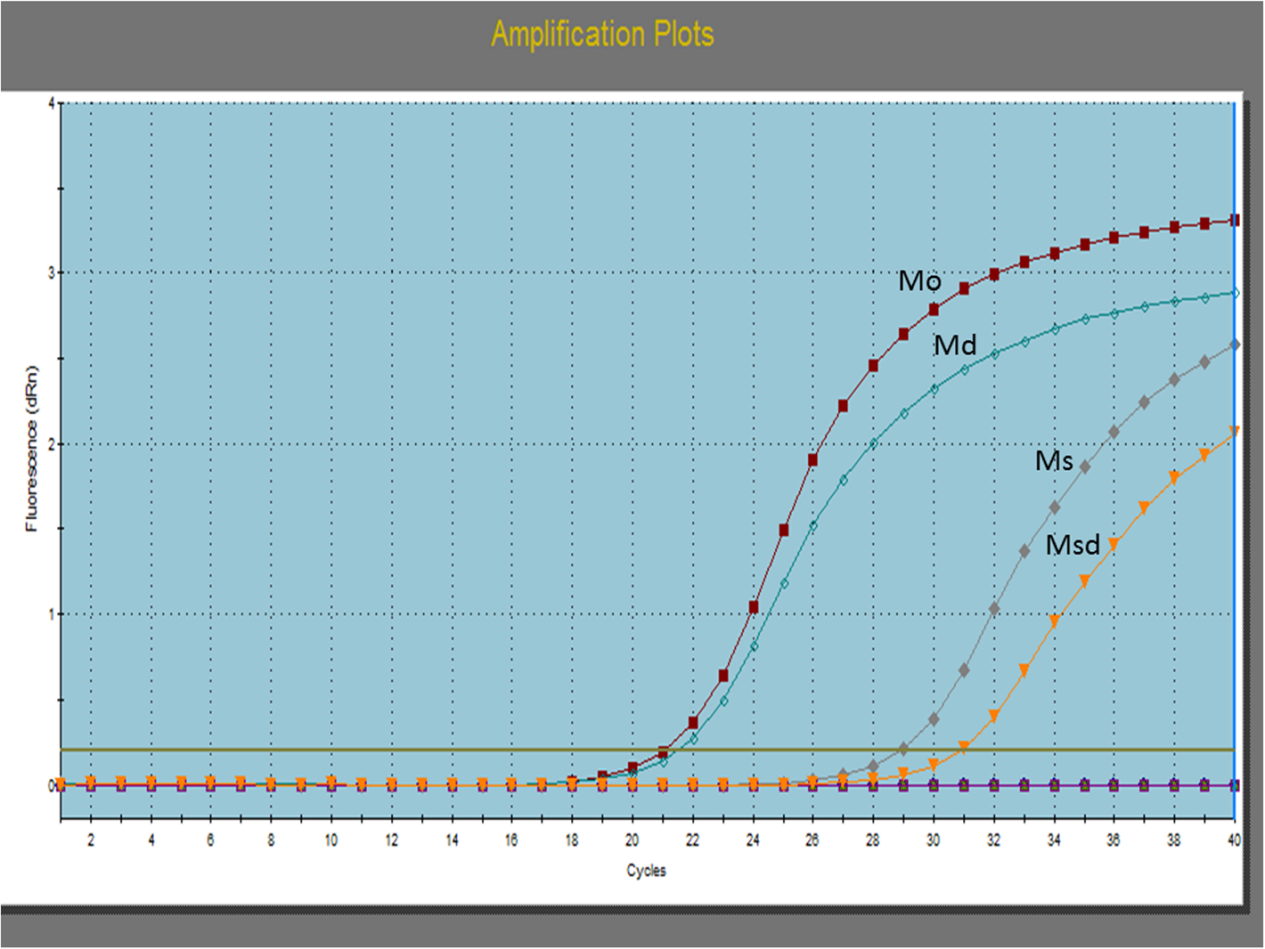
Amplification curve. M0: DNA without digestion. Ms: methylation-sensitive restriction enzyme used. Md: using methylation-dependent Msd: double restriction endonuclease digestion

Quantitative polymerase chain reaction (qPCR) for methylation status using (Max3005P QPCR system; Stratagene, Agilent Technologies, CA) was performed as follows: the enzyme reactions were mixed directly with qPCR master mix (RT^2^ qPCR SYBR Green/ROX Master Mix, Cat number 330520) and were dispensed into a PCR plate containing pre-aliquoted primer mixes (EpiTect Methyl II qPCR Primer Assay Cat number 335002, PTEN Cat number: EPHS101755-1A. The PCR conditions were 95 °C for 10 min (1 cycle), then 99 °C for 30 s and 72 °C for 1 min (3 cycles). Finally, 97 °C for 15 s and 72 °C for 1 min (40 cycles), amplification curve was plotted in Fig. 1. When the cycling program had finished, the raw ΔCT values were obtained and the proportional quantity of methylated and unmethylated DNA portions was assessed by the EpiTect Methyl II PCR Array Microsoft Excel-based data analysis template (www.sabiosciences.com/dna_methylation_data_analysis.php).

### Statistical analysis

Statistical analyses were done using SPSS package software version 16.0. Two-tailed *P* value was significant at *P* < 0.05. Receiver operating characteristic (ROC) curve was made to assign the best cutoff point that amplifies the sensitivity and specificity sum for the assayed gene and to identify its cutoff point that differentiates among tumor, benign, and control groups (non-tumor) [36]. ANOVA and chi-square tests were done as appropriate. Spearman rank correlation was used to assess correlations between studied markers. Prognostic effect as progression-free survival (PFS) was evaluated by establishing the time from the first obtaining neoadjuvant treatment plan to local or distal repetition, contralateral BC, or second primary cancer, whereas overall survival (OS) was appraised from the date of initial diagnosis to the date of patient’s last follow-up or death. Survival distributions were determined by the Kaplan-Meier analysis.

## Results

In the current study, individuals were categorized according to their clinicopathological criteria into breast cancer group (*n* = 112) (classified into 66 patients with IDC and 46 patients with DCI) as these were the types of breast cancer present while collecting samples, benign breast lesions (*n* = 41), and control group (*n* = 25), clinical and demographic factors were reported in Table 1.

No significant difference was detected between age among different studied group control, benign breast lesion, and breast cancer group (*F* = 2.92, *P* = 0.07); also, no significance was reported regarding menopausal status among the studied groups (*X*^2^ = 0.232, *P* = 0.63).

### Mean levels and positivity rates for investigated items among enrolled groups

**Fig. 2:**
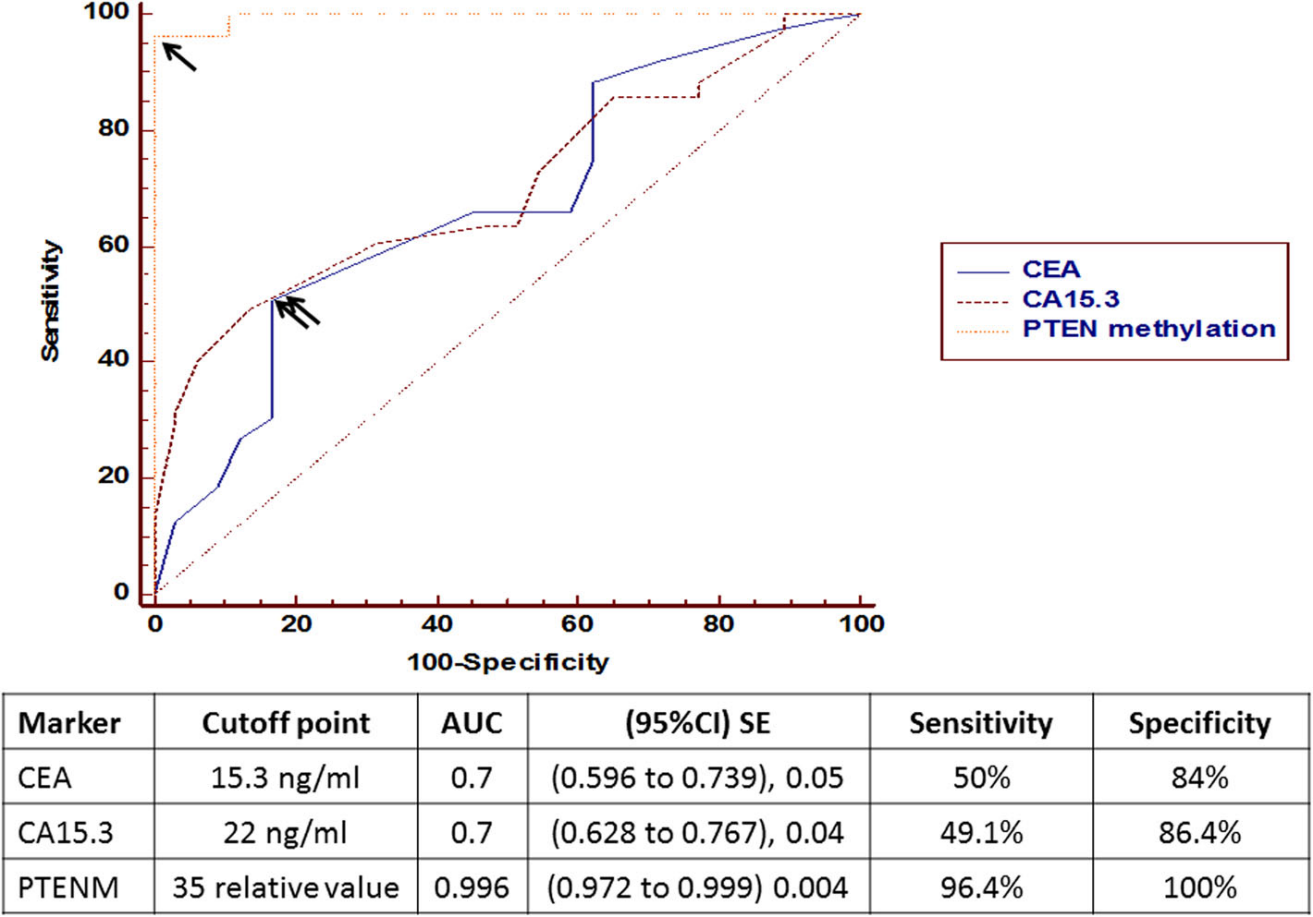
Receiver operating characteristic (ROC) curve for investigated markers

As shown in Table 2, mean levels for investigated tumor markers and methylation pattern of PTEN were significantly increased in the breast cancer group as compared to benign and control groups. Also to detect the diagnostic efficacy receiver operating characteristic curve was plotted for the investigated markers, Fig. 2 which revealed the superiority of PTEN methylation status as a good discriminator from cancer and non-cancer (benign and control) groups with its highest AUC and increased sensitivity and specificity over tumor markers. By considering the cutoff point for each of them, the positivity rates (values above the cutoff points) were detected and reported to be higher in PTEN as reported in Table 2.

**Table 2:** Distribution of mean levels and positivity rates for tumor markers and PTEN methylation status

Markers	Breast cancer	Benign breast lesion	Control individuals
CEA (ng/ml)			
Mean ± SEM	15.8 ± 0.5	12.6 ± 0.6	11.3 ± 0.7
Positivity rate	49.1%	12.2%	0%
Statistics	*F* = 11.5, *P* = 0.001, *X*^2^ = 15.6, *P* < 0.001		
CA15.3 (ng/ml)			
Mean ± SEM	22.2 ± 0.8	17 ± 1	10.6 ± 0.3
Positivity rate	50%	14.6%	0%
Statistics	*F* = 13.5, *P* < 0.001, *X*^2^ = 17.1, *P* < 0.001		
PTEN methylation status %			
Mean ± SEM	87 ± 0.6	25 ± 0.7	12.6 ± 0.3
Positivity rate	96.4%	0%	0%
Statistics	*F* = 350, *P* < 0.001, *X*^2^ = 15.6, *P* < 0.001

### Impact of investigated markers on clinicopathological factors

No significance was reported between tumor markers (CEA and CA15.3) among clinicopathological factors, while a significant difference was reported between the mean level of PTEN methylation status and clinicopatholgical factors as summarized in Table 3. PTEN hypermethylation was reported with advanced criteria of breast cancer as those with IDC, late clinical stage (II–III), high grade tumors (III), and positive lymph node involvement reported increased significant methylation status, as compared to DCI, early stage (0–I), low grade tumors (I–II), and negative lymph node involvement respectively (*P* < 0.001).

**Table 3:** Mean level of PTEN methylation status among clinicopathological factors

Characters	PTEN methylation status
	Mean ± SEM
Age	
≤ 50 years	84 ± 1.8
> 50 years	71.7 ± 2.6
	*F* = 14.9, *P* < 0.001
Menopausal status	
Pre-menopause	83.7
Post-menopause	68.9
	*F* = 21.9, *P* < 0.001
Pathological type	
DCI	66.5
IDC	86.1
	*F* = 46.5, *P* < 0.001
Clinical stage	
Early stage	67.7
Late stage	85.8
	*F* = 46.5, *P* < 0.001
Histological grade	
Low grade	67.7
High grade	84.4
	*F* = 32.5, *P* < 0.001
Lymph node involvement	
Negative	71.5
Positive	85.5
	*F* = 20.3, *P* < 0.001
ER status	
Negative (*n*)	73.7
Positive (*n*)	82.7
	*F* = 7.5, *P* = 0.007
PgR status	
Negative (*n*)	72.9
Positive (*n*)	80.8
	*F* = 5.3, *P* = 0.023
HER-2neu status	
Negative (*n*)	70.8
Positive (*n*)	82
	*F* = 11.3, *P* = 0.001

Methylation levels of PTEN were higher in the blood of patients with ER-positive than ER-negative breast cancers (*P* = 0.007), Pgr positive vs Pgr negative (*p* = 0.023) and in HER2 positive vs. HER2 negative tumors (*P* = 0.001).

A significant correlation between PTEN methylation status and CA15.3 was reported when considering breast cancer patients as reported in Table 4.

**Table 4:** Correlation between PTENM status and tumor markers in breast cancer group (*n* = 112)

	PTEN methylation status	
	*R*	*P*
CEA	0.181	0.057
CA15.3	0.24	0.011

### Relation between PTEN methylation status and survival criteria of breast cancer

**Fig. 3:**
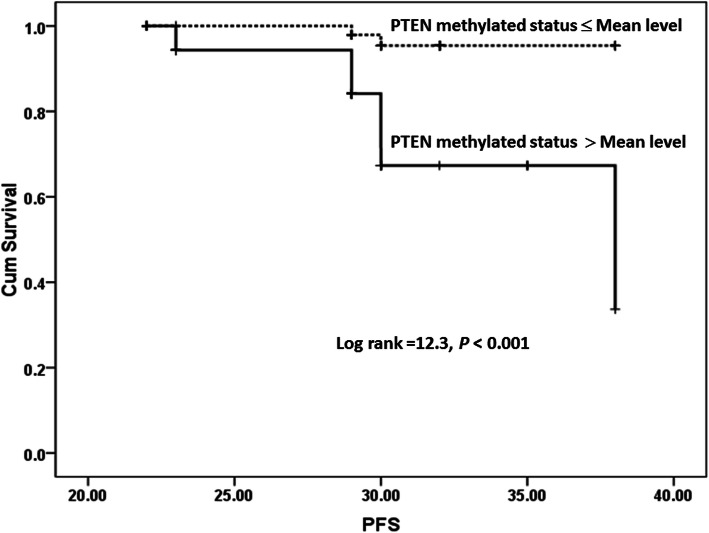
Progression-free survival of BC patients for PTEN methylation

**Fig. 4:**
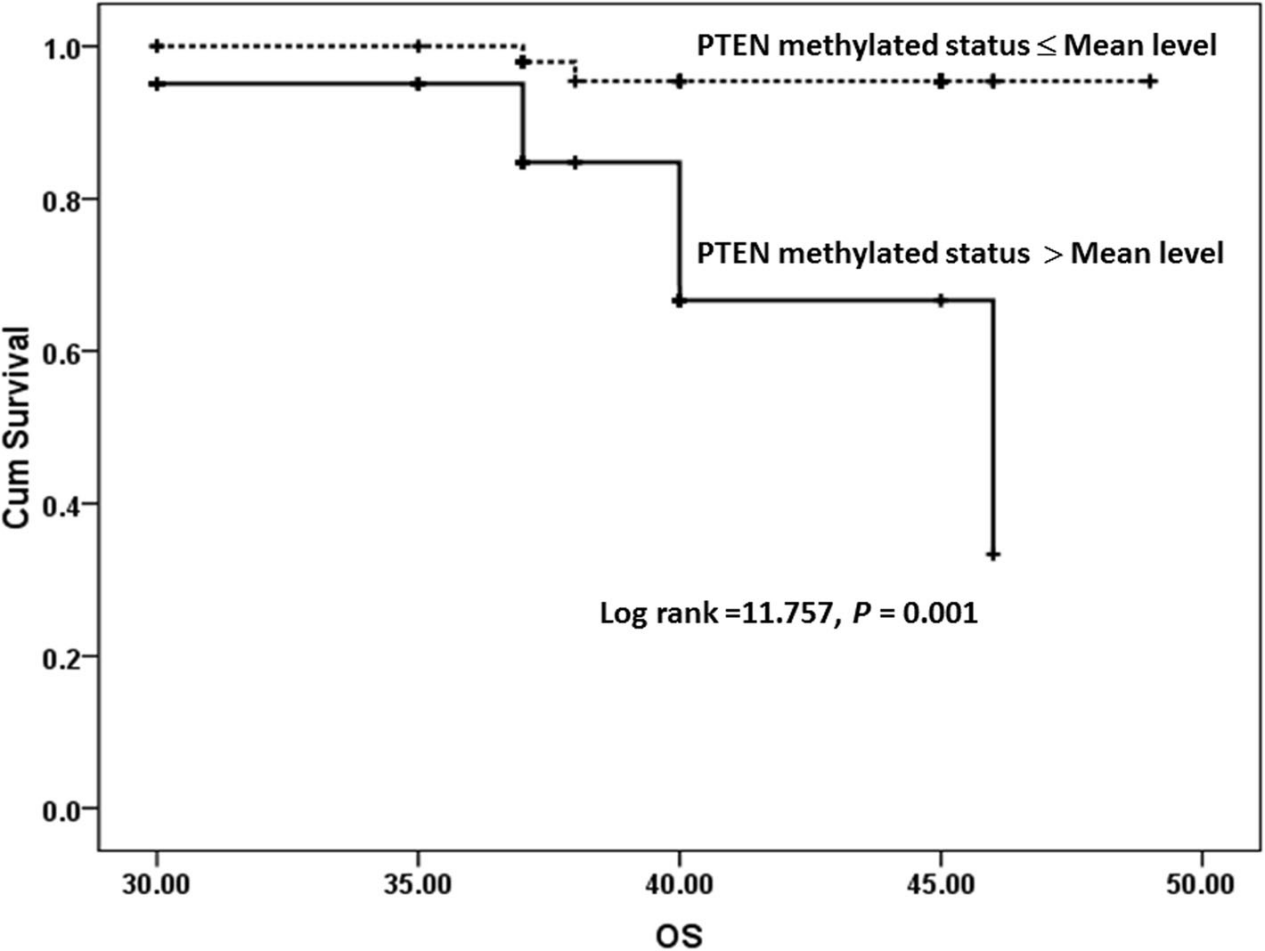
Overall survival for PTEN methylation in BC patients

Breast cancer patients were followed up for 40 months (nearly 3 years); accordingly, their survival patterns with PTEN methylation were studied. The mean level of PTEN methylation status (77 relative value) was selected to categorize breast cancer groups into two groups. As reported in Table 5 and Figs. 3 and 4 significant difference was revealed between PTEN methylation status and worse PFS and OS.

**Table 5:** Survival analysis of breast cancer patients according to PTEN methylation status

PTEN methylation status	Mean				Median			
	Estimate	SE	95% confidence interval		Estimate	SE	95% confidence interval	
			Lower bound	Upper bound			Lower bound	Upper bound
PFS analysis								
≤ 77 (mean level)	37.6	0.27	37.08	38.14	38	5.77	26.68	49.32
> 77 (mean level)	34.89	0.92	33.08	36.7				
Overall	36.5	0.42	35.68	37.34				
OS analysis								
≤ 77 (mean level)	48.47	0.36	47.76	49.188	46	4.34	37.49	54.5
> 77 (mean level)	43.19	0.81	41.6	44.789				
Overall	46.83	0.57	45.715	47.95

## Discussion

Hormone receptors, tumor burden, HER-2, and Ki-67 levels are from tumor biopathological elements which are used as prognostic factors for BC [37, 38]. All these factors should be determined by biopsy or procedure directly from tumor tissue. It would be in demand to use circulating markers as accessible factors for prognosis. Currently, its lower sensitivity and specification have limited the use of serum tumor markers in BC. Many researches have declared a low positive value of CA15-3 and an even lower value of CEA [39, 40]. With no more potent serum markers, even if deficient, CA15-3 and CEA persist the widely used breast cancer biomarkers and are approved by the American Society of Clinical Oncology (ASCO) for practical use. But, due to inadequate data, the use of CA15-3 and CEA as inspection, diagnostic, and stage testing to observe repetition and track the treatment response is not advisable by ASCO furthermore its prognostic purpose [41]. Nevertheless, the European Group on Tumor Markers has suggested their use to evaluate the prognosis in breast cancer [42].

Despite the impressive advances in treatment that have been offered by individualized therapy, breast cancer continues as a major prevalent women’s malignancy, so it takes the greatest public health concern. So, new dependable markers for prognosis are required to distinguish those patients having a high possibility of disease repetition, and who would consequently profit from extra offensive adjuvant therapy and/or nearer follow-up.

The level of DNA methylation in the promoter region of tumor suppressor genes, transcription factors, and drug response genes may perform a function in the inception of cancer, tumor evolution, and treatment comeback [43]. Lately, research of breast cancer has been interested in the recognition of tumor-related biomarkers that can aid in diagnosis steps, therapeutical strategy, and prognosis [44]. Identifying early epigenetic alterations as epimarkers of breast cancer might provide useful indicators for early discovery and help to explain how these alterations impact the disease evolution and the patient’s outcome [43].

In the present study, the authors studied the levels of investigated tumor markers (CEA and CA15.3) using an ELISA kit and the methylation pattern of PTEN using EpiTect Methyl II PCR system. The EpiTect Methyl II PCR system which uses MethylScreenTM technology is a new technology that allows easy, fast, and accurate screening of CpG island DNA methylation for a large number of genes or samples at the same time without bisulfite conversion. The results showed that mean levels of the tumor markers (CEA and CA15.3) were significantly increased in blood samples of breast cancer patients as compared to the benign and control groups and the mean level of PTEN methylation increased significantly in BC patients, these findings proved that the frequency of PTEN methylation was significantly higher in breast cancer patients than in corresponding benign cases and control group (*p* < 0.001) which proposes that PTEN methylation might have a significant role in breast cancer developing. These results were in line with the results of Barekati et al. [45] who examined PTEN promoter methylation in three paralleled samples from BC, inclusive of malignant tissue, normal matched neighboring tissue, and serum blood samples, and they declared that PTEN methylation degrees were increased significantly in serum and tumor tissue of patients compared to those in the normal breast tissue. As regards the diagnostic efficacy, PTEN methylation status revealed the superiority as a good discriminator between cancer and non-cancer groups with its highest AUC and increased sensitivity (96.4%) and specificity (100%) over tumor markers (50% and 84% for CEA and 49.1% and 86.4% for CA15.3), respectively. PTEN methylation pattern was detected in the blood of 112 breast cancer patients, 41 benign cases, and 25 healthy controls and compared between them. The results showed that the frequency of PTEN methylation is 96.4% of BC patients and none of the benign patients and controls showed PTEN methylation. These findings were in concordance with many studies. Zhang et al. [24] recorded a frequency of 31.1% for PTEN methylation in Chinese patients with breast cancer. Also, a previous study found that patients with breast invasive ductal carcinoma show PTEN methylation in 34% of cases [21]. It has been pointed that PTEN inactivation in BC may result from the methylation of the PTEN promoter [24]. Soria et al. [46] revealed PTEN methylation in 35% of patients with non-small cell lung cancer (NSCLC ) and 69% of NSCLC cell lines. Khan et al. [21] stated that PTEN methylation occurred in 34% of breast cancers, and loss of PTEN protein was revealed in 60% of these cases. Yin et al. [47] stated that PTEN gene inactivation in sarcomas of soft tissue may be consequent to hypermethylation in its promoter. The aberrant methylation of the CpG islands within the PTEN promoter may employ as a prospective epigenetic marker for soft tissue sarcoma (STSs). Garcia et al. [48] stated that PTEN promoter hypermethylation may be a potential mechanism for sporadic breast cancer which correlates with other prognostic factors of this cancer. Also, in tumors with aberrant hypermethylation, PTEN expression was lower. Variances between rates of PTEN promoter methylation in this study and some other studies may be due to lifestyle disparities, ethnic origin, and carcinogenic exposure of the inhabitants that could affect in the promoter methylation rate [49].

The current study evaluated the correlations of PTEN methylation with the clinicopathological factors and breast cancer prognosis. Our findings provide a significant positive correlation between PTEN methylation and advanced criteria of breast cancer, including involvement of lymph nodes (*p* < 0.001), later clinical stage (II–III), (*p* < 0.001), and high-grade tumors (III) (*p* < 0.001). Our results are in line with findings of Zakia Kazim in Indian breast cancer patients [50]. The methylation status of the PTEN promoter was studied by Alam et al. [51]. Promoter methylation was seen in 58.5% of breast carcinoma cases from the Saudi population. A significant correlation was observed between PTEN promoter methylation and tumor grade and stage. These results indicate that promoter methylation of the PTEN gene is linked with advanced stage and higher grade of the disease.

Due to the heterogeneity of breast cancer, we also decided to study the correlations between PTEN methylation and the molecular subtypes of breast cancer. The current results showed that PTEN methylation was significantly related with positive ER expression (*p* = 0.007) and positive HER-2 expression (*p* = 0.001) which in concordance with Kaljic et al. [43]. All of these findings reinforce the theory that PTEN has a significant role in the suppression of breast cancer and the thought that PTEN methylation is implicated in malignant initiation and progression. Our results were in agreement with that of Zhang et al. [24] who assessed PTEN promoter hypermethylation in ER-positive and ER-negative BC and revealed a high frequency of hypermethylation in ER-positive cases. Also, Klajic et al. [43] declared that *z*-scores of *PTEN* promoter hypermethylation in HER-2-positive cases were significantly higher than that in HER-2-negative BC.

In the current study, the relations of PTEN methylation with the survival outcomes of breast cancer patients were evaluated. The findings of our study showed that PTEN methylation was significantly linked with the poor progression-free survival (PFS) and worse overall survival (OS) (*P* < 0.001 for both). These results agreed with that of Xu et al. [52] who found that the lowered PTEN expression was significantly associated with the overall survival (OS) and the disease-free survival (DFS) of patients.

## Conclusions

PTEN methylation may foretell more aggressive behavior and worse outcomes in breast cancer patients and could provide helpful prognostic information during the treatment of breast cancer. Moreover, the current study focuses on the prognostic role of PTEN among Egyptian individuals with breast cancer and emphasizes their importance as diagnostic efficacy between non-cancer individuals and cancer patients. Moreover, a significant relation was reported between PTEN methylation status and survival pattern, a future study is ongoing on a bigger Egyptian cohort with different breast cancer subtypes to focus on this finding which may be related to the genetic predisposition of Egyptian patients

## Data Availability

All data generated or analyzed during this study are included.

## Abbreviations

IDC: Invasive duct carcinoma
DCI: Duct carcinoma in situ
BC: Breast cancer
PTEN: Phosphatase and tensin homolog gene
HER-2neu status: Human epidermal growth factor receptor-2
ER status: Estrogen receptor
PgR status: Progesterone receptor
QPCR: Real-time polymerase chain reaction

## References

[1] Jemal A, Bray F, Center MM, Ferlay J, Ward E, Forman D (2011). Global cancer statistics. CA Cancer J Clin.

[2] Hanahan D, Weinberg RA (2000). The hallmarks of cancer. Cell.

[3] Esteller M (2011). Cancer epigenetics for the 21st century: what’s next?. Genes Cancer.

[4] Atalay C (2013). Epigenetics in breast cancer. Exp Oncol.

[5] Phuong NTT, Kim SK, Lim SC, Kim HS, Kim TH, Lee KY, Ahn SG, Yoon GH, Kang KW (2011). Role of pten promoter methylation in tamoxifen-resistant breast cancer cells. Breast Cancer Res Treat.

[6] Lubecka-Pietruszewska K, Kaufman-Szymczyk A, Stefanska B, Fabianowska Majewska K (2013). Folic acid enforces DNA methylation mediated transcriptional silencing of pten, apc and rarbeta2 tumour suppressor genes in breast cancer. Biochem Biophys Res Commun.

[7] Conway K, Edmiston SN, May R, Kuan P, Chu H, Bryant C, Tse C (2014). DNA methylation profiling in the carolina breast cancer study define cancer subclasses differing in clinicopathologic characteristics and survival. Breast Cancer Res.

[8] Harahap WA, Sudji IR, Nindrea RD (2018). BRCA1 promoter methylation and clinicopathological characteristics in sporadic breast cancer patients in Indonesia. Asian Pac J Cancer Prev.

[9] Swellam M, Abdelmaksoud MD, Sayed Mahmoud M, Ramadan A, Abdel-Moneem W, Hefny MM (2015). Aberrant methylation of APC and RARβ2 genes in breast cancer patients. IUBMB Life.

[10] Xu X, Gammon MD, Zhang Y, Cho YH, Wetmur JG, Bradshaw PT, Garbowski G, Hibshoosh H, Teitelbaum SL, Neugut AI, Santella RM, Chen J (2010). Gene promoter methylation is associated with increased mortality among women with breast cancer. Breast Cancer Res Treat.

[11] van Hoesel AQ, Sato Y, Elashoff DA, Turner RR, Giuliano AE, Shamonki JM, Kuppen PJ, van de Velde CJ, Hoon DS (2013). Assessment of DNA methylation status in early stages of breast cancer development. Br J Cancer.

[12] Luo S, Chen J, Xianwei Mo X (2016). The association of PTEN hypermethylation and breast cancer: a meta-analysis. Onco Targets Ther.

[13] Wang X, Cao X, Sun R, Tang C, Tzankov A, Zhang J, Manyam GC, Xiao M, Miao Y, Jabbar K, Tan X, Pang Y, Visco C, Xie Y, Dybkaer K, Chiu A, Orazi A, Zu Y, Bhagat G, Richards KL, Hsi ED, Choi WWL, van Krieken JH, Huh J, Ponzoni M, Ferreri AJM, Møller MB, Parsons BM, Winter JN, Piris MA, Li S, Miranda RN, Medeiros LJ, Li Y, Xu-Monette ZY, Young KH (2018). Clinical significance of PTEN deletion, mutation, and loss of PTEN expression in de novo diffuse large B-cell lymphoma. Neoplasia.

[14] Han F, Hu R, Yang H, Liu J, Sui J, Xiang X, Wang F, Chu L, Song S (2016). PTEN gene mutations correlate to poor prognosis in glioma patients: a meta-analysis. Onco Targets Ther.

[15] Shearn CT, Petersen DR (2015). Understanding the tumor suppressor PTEN in chronic alcoholism and hepatocellular carcinoma. Adv Exp Med Biol.

[16] Cabrita R, Mitra S, Sanna A, Ekedahl H, Lövgren K, Olsson H, Ingvar C, Isaksson K, Lauss M, Carneiro A, Jönsson G (2020). The role of PTEN loss in immune escape, melanoma prognosis and therapy response. Cancers (Basel).

[17] Beg S, Siraj A, Jehan Z (2015). PTEN loss is associated with follicular variant of Middle Eastern papillary thyroid carcinoma. Br J Cancer.

[18] El Kholy MA, El Kholy EA (2018). Endometrial hyperplasia versus carcinoma: does phosphatase and tensin homolog immunohistochemical expression differentiate between them. Sci J Al-Azhar Med Fac Girls.

[19] Li S, Shen Y, Wang M, Yang J, Lv M, Li P, Chen Z, Yang J (2017). Loss of PTEN expression in breast cancer: association with clinicopathological characteristics and prognosis. Oncotarget.

[20] Shetty PJ, Pasupuleti N, Chava S, Nasaruddin K, Hasan Q (2011). Altered transcription and expression of PTEN in breast tumors: is it regulated by hypermethylation?. Breast Dis.

[21] Khan S, Kumagai T, Vora J, Bose N, Sehgal I, Koeffler PH, Bose S (2004). PTEN promoter is methylated in a proportion of invasive breast cancers. Int J Cancer.

[22] Tserga A, Michalopoulos NV, Levidou G, Korkolopoulou P, Zografos G, Patsouris E, Saetta AA (2012). Association of aberrant DNA methylation with clinicopathological features in breast cancer. Oncol Rep.

[23] Jones N, Bonnet F, Sfar S, Lafitte M, Lafon D, Sierankowski G, Brouste V, Banneau G, Tunon de Lara C, Debled M, MacGrogan G, Longy M, Sevenet N (2013). Comprehensive analysis of PTEN status in breast carcinomas. Int J Cancer.

[24] Zhang HY, Liang F, Jia ZL, Song ST, Jiang ZF (2013). Pten mutation, methylation and expression in breast cancer patients. Oncol Lett.

[25] Downs BM, Mercado-Rodriguez C, Cimino-Mathews A, Chen C, Yuan JP, Van Den Berg E, Cope LM, Schmitt F, Tse GM, Ali SZ, Meir-Levi D (2019). DNA methylation markers for breast cancer detection in the developing world. Clin Cancer Res.

[26] Pu RT, Laitala LE, Alli PM, Fackler MJ, Sukumar S, Clark DP (2003). Methylation profiling of benign and malignant breast lesions and its application to cytopathology. Mod Pathol.

[27] Wu L, Shen Y, Peng X, Zhang S, Wang M, Xu G, Zheng X, Wang J, Lu C (2016). Aberrant promoter methylation of cancer-related genes in human breast cancer. Oncol Lett.

[28] Yari K, Payandeh M, Rahimi Z (2016). Association of the hypermethylation status of PTEN tumor suppressor gene with the risk of breast cancer among Kurdish population from Western Iran. Tumor Biol.

[29] Xu Z, Sandler DP, Taylor JA (2020). Blood DNA methylation and breast cancer: a prospective case-cohort analysis in the sister study. JNCI J Natl Cancer Inst.

[30] Ezzat GM, El-Shoeiby MH (2019). Determinants of *p14/ARF* methylation in healthy females: association with reproductive and non-reproductive risk factors of breast cancer. Egypt J Med Hum Genet.

[31] Yadav P, Masroor M, Nandi K, Kaza RCM, Jain SK, Khurana N, Saxena A (2018). Promoter methylation of BRCA1, DAPK1 and RASSF1A is associated with increased mortality among Indian women with breast cancer. Asian Pac J Cancer Prev.

[32] Greene FL, Sobin LH (2008). The staging of cancer: a retrospective and prospective appraisal. CA Cancer J Clin.

[33] Robbins P, Pinder S, de Klerk N, Dawkins H, Harvey J, Sterrett G, Ellis I, Elston C (1995). Histological grading of breast carcinomas: a study of interobserver agreement. Hum Pathol.

[34] Phillips T, Murray G, Wakamiya K, Askaa J, Huang D, Welcher R, Kurt Pii K, Allred DC (2007). Development of standard estrogen and progesterone receptor immunohistochemical assays for selection of patients for antihormonal therapy. Appl Immunohistochem Mol Morphol.

[35] Iqbal BM, Buch A (2016). Hormone receptor (ER, PR, HER2/neu) status and proliferation index marker (Ki-67) in breast cancers: their onco-pathological correlation, shortcomings and future trends. Med J DY Patil Univ.

[36] Zweig MH, Campbell G (2013). Receiver-operating characteristic (ROC) plots: a fundamental evaluation tool in clinical medicine. Clin Chem.

[37] Elston CW, Ellis IO, Pinder SE (1999). Pathological prognostic factors in breast cancer. Crit Rev Oncol Hematol.

[38] Isaacs C, Stearns V, Hayes DF (2001). New prognostic factors for breast cancer recurrence. Semin Oncol.

[39] Shao Y, Sun X, He Y, Liu C, Liu H (2015). Elevated levels of serum tumor markers CEA and CA153 are prognostic parameters for different molecular subtypes of breast cancer. PLoS One.

[40] Wu SG, He ZY, Zhou J, Sun JY, Li FY, Lin Q, Guo L, Lin HX (2014). Serum levels of CEA and CA15-3 in different molecular subtypes and prognostic value in Chinese breast cancer. Breast.

[41] Harris L, Fritsche H, Mennel R, Norton L, Ravdin P, Taube S, Somerfield MR, Hayes DF, Bast RC Jr, American Society of Clinical Oncology (2007) American Society of Clinical Oncology 2007 update of recommendations for the use of tumor markers in breast cancer. 25(33):5287–5312. 10.1200/jco.2007.14.2364 10.1200/JCO.2007.14.2364 17954709

[42] Molina R, Barak V, van Dalen A, Duffy MJ, Einarsson R, Gion M, Goike H, Lamerz R, Nap M, Sölétormos G, Stieber P (2005). Tumor markers in breast cancer—European Group on Tumor Markers recommendations. Tumor Biol.

[43] Klajic J, Fleischer T, Dejeux E, Edvardsen H, Warnberg F, Bukholm I, Lonning PE (2013). Quantitative DNA methylation analyses reveal stage dependent DNA methylation and association to clinicopathological factors in breast tumors. BMC Cancer.

[44] Alipour M, Zargar SJ, Safarian S, Fouladdel S, Azizi E, Jafargholizadeh N (2013). The study of DNA methylation of bax gene promoter in breast and colorectal carcinoma cell lines. Iran J Cancer Prev.

[45] Barekati Z, Radpour R, Kohler C, Zhang B, Toniolo P, Lenner P, Lv Q, Zheng H, Zhong XY (2010). Methylation profile of TP53 regulatory pathway and mtDNA alterations in breast cancer patients lacking TP53 mutations. Hum Mol Genet.

[46] Soria JC, Lee HY, Lee JI, Wang L, Issa JP, Kemp BL, Liu DD, Kurie JM, Mao L, Khuri FR (2002). Lack of pten expression in non-small cell lung cancer could be related to promoter methylation. Clin Cancer Res.

[47] Yin L, Cai WJ, Liu CX, Chen YZ, Hu JM, Jiang JF, Li HA (2013). Analysis of pten methylation patterns in soft tissue sarcomas by MassARRAY spectrometry. PLoS One.

[48] García JM, Silva J, Peña C, Garcia V, Rodríguez R, Cruz MA, Cantos B, Provencio M, España P, Bonilla F (2004). Promoter methylation of the pten gene is a common molecular change in breast cancer. Genes Chromosom Cancer.

[49] Izadi P, Noruzinia M, Karimipoor M, Karbassian MH, Akbari MT (2012). Promoter hypermethylation of estrogen receptor alpha gene is correlated to estrogen receptor negativity in Iranian patients with sporadic breast cancer. Cell J.

[50] Kazim Z, Wahabi K, Perwez A, Lal P, Rizvi MA (2019). PTEN genetic and epigenetic alterations define distinct subgroups in north Indian breast cancer patients. Asian Pac J Cancer Prev.

[51] Alam MS, Jerah ABA, Ashraf AM, Kumaresan K, Eisa ZM, Mikhall NT (2017). Promoter methylation and loss of expression of PTEN gene in breast cancer patients from Saudi population. J Clin Exp Oncol.

[52] Xu F, Zhang C, Cui J, Liu J, Li J, Jiang H (2017). The prognostic value and potential drug target of phosphatase and tensin homolog in breast cancer patients: a meta-analysis. Medicine (Baltimore).

